# Pulsed electromagnetic fields for postmenopausal osteoporosis and concomitant lumbar osteoarthritis in southwest China using proximal femur bone mineral density as the primary endpoint: study protocol for a randomized controlled trial

**DOI:** 10.1186/s13063-015-0780-4

**Published:** 2015-06-10

**Authors:** Hui-Fang Liu, Hong-Chen He, Lin Yang, Zhou-Yuan Yang, Ke Yao, Yuan-Chao Wu, Xi-Biao Yang, Cheng-Qi He

**Affiliations:** Department of Rehabilitation Medicine, West China Hospital, Sichuan University, No. 37, Guo Xue Xiang, Chengdu, People’s Republic of China; Rehabilitation Key Laboratory of Sichuan Province, West China Hospital, Sichuan University, Chengdu, People’s Republic of China; Department of Orthopedics, West China Hospital, Sichuan University, Chengdu, People’s Republic of China; Department of Biostatistics, West China School of Public Health, Sichuan University, Chengdu, People’s Republic of China; Department of Radiology, West China Hospital, Sichuan University, Chengdu, People’s Republic of China

**Keywords:** Menopause, Pulsed electromagnetic fields, Osteoporosis, Osteoarthritis

## Abstract

**Background:**

Osteoporosis (OP) and osteoarthritis (OA) are prevalent skeletal disorders among postmenopausal women. Coexistence is common especially that of postmenopausal osteoporosis (PMO) and lumbar OA. An hypothesis has been raised that OP and OA might share the same pathogenic mechanism, and pulsed electromagnetic fields (PEMFs) were reported to have anti-osteoporosis and anti-osteoarthritis properties, but this suggestion was based primarily on biomarker data. Therefore, whether these two effects could take place simultaneously has not yet been investigated. This randomized controlled trial (RCT) is designed to explore the effect of PEMFs for PMO and concomitant lumbar OA.

**Methods/Design:**

The study will include PMO patients (postmenopausal women; aged between 50 and 70 years; have been postmenopausal for at least 5 years and diagnosed with OP using proximal femur T-score) with concomitant lumbar OA (patients with confounding disorders like diabetes, hypertension, hyperlipidemia, and previous fracture history, *etcetera*, will be excluded) will be randomly assigned to two arms: PEMFs group and sham PEMFs group. There will be 25 participants in each arm (50 in total) and the outcome assessment, including the primary endpoint (proximal femur bone mineral density), will be performed at 5 weeks, 3 months and 6 months after enrollment.

**Discussion:**

PMO and lumbar OA are prominent public health problem, especially for postmenopausal women. We hope this RCT will provide scientific evidence to primary care of the postmenopausal women regarding the use of these nonpharmaceutical, noninvasive modalities, PEMFs, in managing PMO and lumbar OA.

**Trial registration:**

Chinese Clinical Trial Registry: ChiCTR-TRC-14005156 (28 August 2014).

**Electronic supplementary material:**

The online version of this article (doi:10.1186/s13063-015-0780-4) contains supplementary material, which is available to authorized users.

## Background

Osteoporosis (OP) is characterized by low bone mass and microarchitectural deterioration of bony tissue, with a consequent increase in bone fragility and risk of fractures [1]. This systemic skeletal disease is clinically diagnosed by evaluating bone mineral density (BMD) using dual-energy X-ray absorptiometry (DXA) according to the WHO criteria [2]. Another prevalent skeletal disorder among postmenopausal women is osteoarthritis (OA). It involves a series of pathological changes not only in articular cartilage but also the underlying bone [3], which will probably increase average BMD and interfere with the capability of DXA in detecting OP.

In clinical practice, proximal femur and lumbar spine are the two most common sites measured for BMD assessment. For the former, the most vulnerable region to hip OA is the femur head, which is not included in the measurement, so BMD values of proximal femur are believed to be accurate to some extent. The spine is also susceptible to OA, and spinal OA affects almost 80 % of those aged 40 or above [4]. More specifically, since the thoracic segments are “splinting” by the costovertebral joints, and the cervical spine has relatively low need for weight-bearing, the lumbar segments are regarded as the most susceptible region of the spine to OA [5]. So the coexistence of OP and lumbar OA is common and the diagnosis of OP was suggested to be made based on values of hip BMD rather than that of lumbar spine, unless lumbar OA was not present [6, 7].

On the other hand, the relationship between OP and OA is complicated and controversial [8, 9]. One hypothesis is that they might share the same pathogenic mechanism since it was found that excessive bone resorption, a hallmark of OP, also took place in the early stage in the development of OA. This finding and hypothesis are inspiring, since they indicate there might be treatments that could be used to treat OP and OA at the same time [9–12].

Pulsed electromagnetic fields (PEMFs) might be classified as these treatments, because both anti-osteoporosis and anti-osteoarthritis effects have been reported respectively, but primarily with biomarker data [13–24]. No clinical research has ever been conducted to explore the anti-osteoporosis and anti-osteoarthritis effects of PEMFs simultaneously, especially using clinical data. In the clinical setting, the coexistence of postmenopausal osteoporosis (PMO) and lumbar OA is common, and if treatments could be applied to cope with these two problems at the same time, such a treatment would be significant. The primary goal of this randomized controlled trial (RCT) is to explore these two effects by treating PMO (diagnosed by evaluating BMD of proximal femur) with concomitant lumbar OA in southwest China.

## Methods/Design

### Study design

**Fig. 1:**
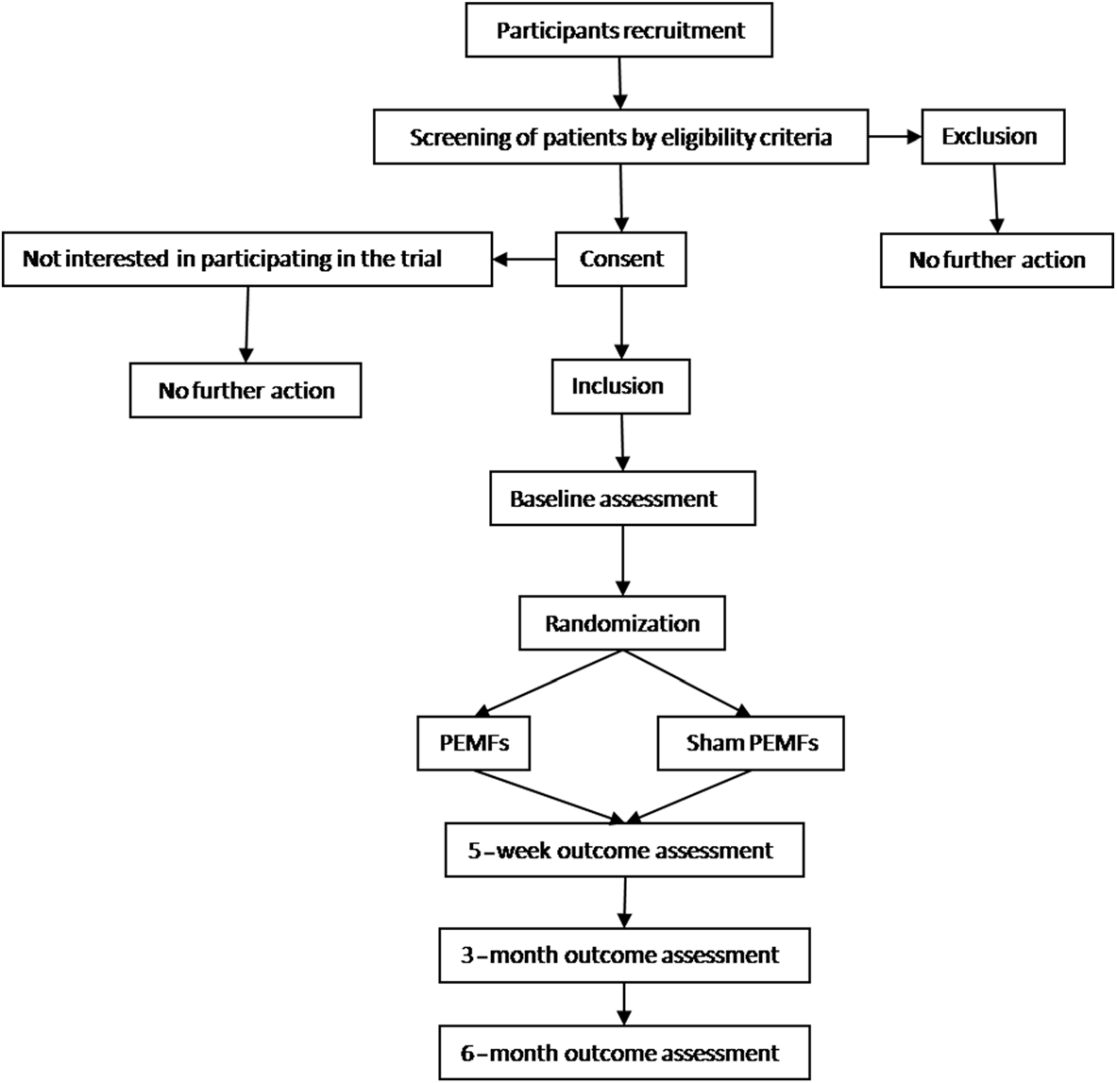
Trial flow. PEMFs, pulsed electromagnetic fields

The research will be a prospective parallel, single-blinded (assessor and participants) RCT with a follow-up period of 6 months. After baseline assessment, participants will be randomized into two arms: PEMFs group and sham PEMFs group (Fig. 1). This randomization process will be automatically performed using software and sequential sealed envelopes by an independent statistician once a patient will be considered eligible. The sealed envelopes will be opened for each participants, and the statistician will make a record of the allocation.

Participants will receive PEMFs or sham PEMFs treatment for the first 5 weeks after enrollment. Outcome assessment will be performed at the following time points: 5 weeks, 3 months and 6 months after enrollment (Additional file 1).

This RCT is planned to be single blinded (assessor and patient). Furthermore, statisticians carrying out the statistical analyses will also be blind to group allocation until the analyses are completed.

This protocol conforms to CONSORT guidelines for nonpharmacological studies [25], in accordance with the ethical standards in the 1964 Declaration of Helsinki and was approved by the clinical research and biomedical ethics committee of the West China Hospital, Sichuan University (ethics reference: 2014 (114)). Written and signed informed consent will be obtained from all participants prior to inclusion in the study. This trial has been registered in the Chinese Clinical Trial Registry (No. ChiCTR-TRC-14005156).

### Participants

To be eligible, participants must meet the following criteria: (1) is a postmenopausal woman; (2) aged between 50 and 70 years; (3) has been postmenopausal for at least 5 years and diagnosed with OP (proximal femur T-score equal to or lower than -2.5) [2, 7]. Women with suspected confounding disorders (for example, diabetes mellitus, hypertension, hyperlipidemia, obesity, secondary and idiopathic osteoporosis, fracture history, systemic inflammatory disorders, visual impairment, severe vestibular lesions, cerebellar lesions, peripheral nerve or muscle disease, mental retardation, mental illness, cognitive impairment, obstetrical and gynecological diseases, blood disease, stroke or venous thromboembolic disease, thyroid, parathyroid, adrenal, hepatic and renal disease, malignancy, *etcetera*), who had received anti-osteoporosis treatment within 6 months before inclusion, and those not suitable to be included for any other reason not mentioned above will all be excluded.

Participants fulfilling the above criteria will receive further lateral lumbar spine radiography, and those with concomitant lumbar OA will finally be included. The diagnosis of lumbar OA will be made when there are two or more criteria fulfilled according to Kellgren and Lawrence [26]. Two technicians will independently read the radiographs and in case of discrepancy, a third technician will be consulted to reach consensus. Further exclusion criteria are as follows: (1) secondary OA; (2) congenital spinal diseases; (3) scoliosis; (4) spondylolisthesis - spondylolysis; (5) history of spinal surgery; (6) inflammatory arthropathy; or (7) individuals who have had facet joint injection or any physical therapy (for example, chiropractic, osteopathy or physiotherapy treatment) for back pain in the previous 6 months.

### Interventions

All included participants will receive conventional usual care (600 mg/day calcium and alfacalcidol vitamin D supplement 0.25 μg/bid) throughout the study period. Participants allocated to the PEMFs group will receive additional PEMFs treatment (40 min/treatment, 1 treatment session/day, 6 treatment sessions/week, for a total of 30 times as one course of treatment) for the first 5 weeks after enrollment, while those assigned to the sham PEMFs group will receive sham PEMFs treatment with the same device but no stimulus generated, indistinguishable from the real ones.

The PEMFs treatment will be applied using XT-2000B therapeutic stimulators (Tianjin xtmed, Tianjin, China) to generate time-varying fields consisting of bursts of asymmetric pulses. Each burst should last for 0.2 ms and repeat at a frequency of 8 Hz. The fields will be delivered perpendicular to the lumbar spine and proximal femur of the supine participant and the flux density within a single burst will start with a peak value of 3.82 mT and decrease to 0 mT in 0.2 ms.

All participants will be asked to refrain from seeking any other physiotherapy or medications throughout the study period, especially during the first 5 weeks after enrollment, when PEMFs are proposed to be delivered. Whereas afterwards, during the follow-up phase when all participants are scheduled to receive only usual care, the use of analgesia and non-steroidal anti-inflammatory drugs (NSAIDs) will be permitted for the sake of ethical considerations. However, the use of this sort of medications should not exceed 3 days in each week and should not occur within 48 h of a follow-up visit. Information regarding the types of treatments and corresponding consumptions will be collected at each visit, and comparability between groups will be tested using Mann-Whitney’s *U*-test.

### Sample size

Because we could not identify data or citations proving references for the minimum clinically significant difference and corresponding standard deviation in proximal femur bone mineral density (BMDF) between the two arms, the “least detectable longitudinal change measured using dual-energy X-ray absorptiometry (DXA), that is, 0.023” was approximately used as the expected absolute intergroup difference and standard deviation [20, 27]. With a type I error probability of 5 % and a 90 % probability of avoiding a type II error, we would require 20 participants in each group. Assuming a loss to follow-up of 20 %, a total sample size of 50 participants (25 participants per group) is needed.

We did additional sample size calculation for pain intensity, the most important secondary endpoint, measured using the Visual Analogue Scale (VAS) on a scale of 0 to 100. With a standard deviation of 32.17 [28], type I error of 5 %, 90 % power, and a 20 % drop-out rate, approximately 25 patients would be needed in each group to show a minimum clinically significant difference, 30-points between groups [29].

### Assessment

The primary outcome will be the mean percentage change in BMDF at 6-month follow-up. Proposed secondary outcomes were listed below: VAS; The mean percentage changes in bone mineral density of the lumbar spine (BMDL); serum 25-hydroxy-vitamin D (25(OH) D) concentrations; Oswestry Disability Index (ODI); manual muscle test (MMT) score; Berg Balance Scale (BBS) score; Timed Up and Go Test (TUG); hemorheological determinants; serum C-terminal telopeptide of type I collagen (sCTX I), serum bone alkalin phosphatase (sBALP), N-MID osteocalcin (N-MID); range of motion (ROM) of lumbar spine; overall functional ability using short form 36 item general health questionnaire (SF-36, Quality Metric, Inc., Lincoln, Rhode Island, USA); treatment compliance; health care consumption costs; possible adverse events (Additional file 1). Detailed information on some of the outcome measurements are described below:

As for BMD measurements, participants will be scanned in the standard manner by a technician using DXA (GE Lunar iDXA with encore 12.0 software, GE Healthcare Lunar, Madison, WI) following recommendations from the manufacturer. For proximal femur, BMD of the total hip will be recorded and analyzed, while for the lumbar spine the average BMD (aBMD) of the four vertebrae (L1-L4) will be extracted. All these data could be directly obtained using corresponding software without further calculations.

Manual muscle strength of the lumbar and the lower-extremity will be estimated by employing the MMT method with the traditional six-point ordinal Medical Research Council scale (0 to 5). Half-point scores will be used between all grades except that of 4 and 5, since it requires manual resistance that cannot be graded reliably [30]. All tests will be performed by a single examiner. Furthermore, before testing, the examiner and participants will all be required to rest for at least 5 min.

The BBS is a set that comprises 14 simple balance related tasks, ranging from standing up from a sitting position, to standing on one foot [31]. Another examiner will perform the tests for all the participants following the standard procedure. The examiner and participants will also be required to rest for at least 5 min before testing.

A TUG test will be conducted by recording the total time that a person takes to rise from a chair, walk 3 meters, turn around, walk back to the chair, and sit down [32]. It is intended to evaluate the functional mobility of the participants.

Adverse events will be recorded throughout the study period. Having once occurred, the intensity and possible relationship with the treatment will be examined and described in detail.

Baseline assessment will be carried out after eligibility and informed consent has been confirmed (Fig. 1). Apart from outcome measurements, information referring to participant’s age (in years), menopause age (in years), time since menopause (in years), body mass index (BMI; in kg/m^2^), comorbidities, duration of low back pain symptoms, medication use, information referring to physical activity (evaluated by the International Physical Activity Questionnaire (IPAQ) short version [33]), sunlight exposure (frequency and duration of time spent outside, sun-protective-factor use), employment status, occupation and dietary intake will all be collected.

### Follow-up

After the first 5 weeks after enrollment, all participants are scheduled to receive only usual care and the research will go into the follow-up phase with scheduled follow-up visits taking place at 5 weeks, 3 months and 6 months after enrollment (Additional file 1). The last two time points are designed to explore possible delayed effects of PEMFs.

### Data analysis

Continuous measurements will be expressed as means and standard deviations (SD) and categorical measurements will be summarized using numbers and percentages. For all variables, descriptive statistics will be calculated.

Our primary analysis of the data will be performed using the principle of intention-to-treat (ITT). All participants with a baseline value and at least one measurement after randomization will be included in the efficacy analyses using the last observation carried forward (LOCF) method. It will consider all participants, including those who are not fully compliant and those with missing outcome data.

In addition, we will conduct sensitivity analysis by conducting a per-protocol (PP) analysis for the primary outcome at 6 months. It will include participants whose compliance is good, which is defined as attendance of all the scheduled visits, and low contamination (no other treatment during the first 5 weeks after enrollment).

#### Descriptive analyses at baseline

Baseline comparability between groups will be tested using Pearson’s chi-square test or Fisher’s exact test for categorical data, and Mann-Whitney’s *U*-test for continuous data. All baseline measurements will be analyzed.

#### Primary analysis

Treatment differences will be assessed on the percentage change from baseline to the three follow-up visits. Our primary analysis will be conducted regarding the effect of PEMFs on BMDF at 6 months. Descriptive statistics for this variable will be presented for each intervention group at each appointment during the 6-month follow-up. Comparisons will be carried out using a mixed linear model [34] with treatment group as a fixed factor and time as a random factor. Adjustments will be made for covariates including participant’s age, menopause age, time since menopause, BMI, and the corresponding baseline value. This model is supposed to be able to appropriately control for correlation among multiple observations. Sensitivity analysis will also be carried out by independent t-tests comparing changes between groups from baseline to the end of follow-up. When intragroup comparison is needed, paired sample *t*-tests will be also applied.

#### Secondary analyses

The analyses for the secondary outcome measurements will be carried out in the same way as those mentioned above. A mixed linear model will be applied, with treatment group as a fixed factor, and time as a random factor, adjusting for similar confounding factors. In addition, to explore relationships among variables, we will calculate Spearman’s or Pearson’s correlation coefficients along with their *P* values.

#### Health economics evaluation

To determine whether the PEMFs are cost-effective to pay for additional health gain, we will compute mean (and SD) per patient costs and incremental cost-utility ratios using quality-adjusted life-years (QALYs) derived from SF-36 scores with appropriate preference weightings [35, 36].

For all the analyses mentioned above, a two-sided value of *P* <0.05 will be considered statistically significant. All statistical analyses will be carried out using 19.0 SPSS software (SPSS, Chicago, IL).

## Discussion

This paper describes the rationale and protocol for conducting a preliminary RCT in southwest China that will investigate the effectiveness of PEMFs in treating PMO and concomitant lumbar OA.

The idea originated from daily clinical practice since the coexistence of OP and OA is not rare, especially that of PMO and lumbar OA, which is frequently found during BMD examinations using DXA. Furthermore, it was found that excessive bone resorption also took place in the early stage in the development of OA, indicating OP and OA might share the same pathogenic mechanism and could be treated simultaneously [9–12]. As for treatment method, PEMFs have been reported to have anti-osteoporosis and anti-osteoarthritis properties, but whether these two effects would take place at the same time has not yet been evaluated. Since the two skeletal disorders, PMO and lumbar OA, are prevalent among postmenopausal women and have a considerable impact on both individuals and the society [37, 38], it would be of great significance to identify effective treatments that could deal with the two clinical problems simultaneously.

This trial will be the first RCT to investigate the effect and cost-utility of PEMFs for PMO and concomitant lumbar OA. The combination of clinical efficacy and cost-effectiveness evidence is essential for health care decisions. To find improvements for both OP and lumbar OA and to facilitate comparability with other research, we will include valid outcome measurements, and to minimize bias, we will use BMDF, not BMDL, for the diagnosis of OP, eliminating the interference of lumbar OA for measuring BMD. Furthermore, we will choose BMD (specifically the mean percentage change in BMDF), instead of the incidence of fractures, as our primary endpoint, because a change in BMD offers the advantages of high precision, the ability to study multiple skeletal sites in the same patient, and a relatively rapid response to treatment [39, 40]. However, patient relevance of any positive result of this trial will require a follow-up trial with patient-relevant endpoints like fracture and health economics evaluations using QALYs and SF-36 scores informed by fracture data, *etcetera*, to provide further evidence.

We acknowledge limitations in the trial. First, the sample size calculation is somewhat imprecise, and there are many secondary outcomes, all at 0.05 significance in a trial with 25 subjects in each arm after accounting for a 20 % withdrawal. The risk of type I error may be very high. Second, the primary endpoint is BMDF, which is not as patient-relevant as fractures, so relevance of any positive result of this trial to patients needs further investigations in large well-designed trials. In spite of these limitations, our research also has strengths. One is that we refined our exclusion criteria to eliminate potential confounding factors as far as possible. We will exclude patients with confounding disorders and concomitant disorders that are of possibility to interfere with the balance of bone metabolism, and patients with lesions that may not be able to fulfill the study assessment. Furthermore, those who received treatments within 6 months before inclusion will also be excluded. Another one is the utilization of a patient-blinded design, with sham PEMFs arm to reduce the risk of bias. It is of great significance especially for the assessment of patient-reported outcomes (for example, pain), since without a proper patient-blinded design, identified differences could be interpreted as the results of placebo effect, which is found effective in the treatment of OA, especially for pain, stiffness and self-reported function [41]. There are other trials that have used sham PEMFs successfully. For example, Shi *et al*. evaluated outcomes of postoperative delayed union of long-bone fractures treated with an early application of PEMF, as compared with a sham-treated control group, where the coil was applied for 8 h/day with a sham signal generator from the same manufacturer [42]; Nelson *et al*. examined the pain-relieving effect of PEMFs and the sham devices they used were activated with a switch and had blinking indicator lights, just as active devices [43]. These two trials had used sham PEMFs successfully, and their findings were convincing.

In summary, this trial will provide preliminary evidence regarding the use of a nonpharmaceutical, noninvasive modality for managing PMO and lumbar OA. The success of this study will provide scientific evidence to primary care of postmenopausal women with these two common skeletal disorders.

### Trial status

The trial is currently in the process of patient recruitment.

## Additional file

Additional file 1:
**Schedule of visits and measures to be made.**

## Abbreviations

BBS: Berg balance scale
BMD: bone mineral density
BMDF: proximal femur bone mineral density
BMDL: bone mineral density of the lumbar spine
BMI: body mass index
DXA: dual-energy X-ray absorptiometry
ITT: intention-to-treat
LOCF: last observation carried forward
MMT: manual muscle test
N-MID: N-MID osteocalcin
NSAID: non-steroidal anti-inflammatory drug
OA: osteoarthritis
ODI: Oswestry disability index
PEMFs: pulsed electromagnetic fields
QALY: quality-adjusted life year
ROM: range of motion
sBALP: serum bone alkalin phosphatase
sCTX I: serum C-terminal telopeptide of type I collagen
SD: standard deviation
SF-36: Short Form 36-item General Health Questionnaire
TUG: Timed Up and Go test
VAS: visual analogue scale
25(OH) D: 25-hydroxy-vitamin D
