# Supplementary material for: Pulsed electromagnetic fields for postmenopausal osteoporosis and concomitant lumbar osteoarthritis in southwest China using proximal femur bone mineral density as the primary endpoint: study protocol for a randomized controlled trial
Source: Trials. 2015 Jun 10;16:265. doi: 10.1186/s13063-015-0780-4 (PMC4482283; doi:10.1186/s13063-015-0780-4)
Supplement: Additional file 1: — Schedule of visits and measures to be made. [file 13063_2015_780_MOESM1_ESM.doc]

Additional file 1

**Table 1 Schedule of visits and measures to be made**

| **Measure** | **Baseline visit** | **5-week visit** | **3-month visit** | **6-month visit** |
| --- | --- | --- | --- | --- |
| **Baseline measure** |  |  |  |  |
| Demographics and medical history | **√** |  |  |  |
| Background information | **√** |  |  |  |
| **Primary outcome measure** |  |  |  |  |
| Proximal femur bone mineral density (BMDF) | **√** | **√** | **√** | **√** |
| **Secondary outcome measure** |  |  |  |  |
| Visual analogue scale (VAS) | **√** | **√** | **√** | **√** |
| Bone mineral density of the lumbar spine (BMDL) | **√** | **√** | **√** | **√** |
| 25-hydroxy-vitamin D (25(OH) D) concentrations | **√** | **√** | **√** | **√** |
| Oswestry disability index (ODI) | **√** | **√** | **√** | **√** |
| Manual muscle test (MMT) | **√** | **√** | **√** | **√** |
| Berg balance scale (BBS) | **√** | **√** | **√** | **√** |
| Timed Up & Go Test (TUG) | **√** | **√** | **√** | **√** |
| Hemorheological determinants | **√** | **√** | **√** | **√** |
| Serum C-terminal telopeptide of type I collagen (sCTX I) | **√** | **√** | **√** | **√** |
| Serum Bone alkalin phosphatase (sBALP) | **√** | **√** | **√** | **√** |
| N-MID osteocalcin (N-MID) | **√** | **√** | **√** | **√** |
| Range of motion (ROM) of lumbar spine | **√** | **√** | **√** | **√** |
| Short form 36 item general health questionnaire (SF-36) | **√** | **√** | **√** | **√** |
| Treatment compliance | **√** | **√** | **√** | **√** |
| Health care consumption costs | **√** | **√** | **√** | **√** |
| Possible adverse events | **√** | **√** | **√** | **√** |
| **Other outcome measure** |  |  |  |  |
| Concomitant treatment | **√** | **√** | **√** | **√** |
